# Deep learning in time series forecasting with transformer models and RNNs

**DOI:** 10.7717/peerj-cs.3001

**Published:** 2025-07-24

**Authors:** Rogerio Pereira dos Santos, João P. Matos-Carvalho, Valderi R. Q. Leithardt

**Affiliations:** 1 COPELABS, Universidade Lusófona de Humanidades e Technologias, Lisboa, Portugal; 2 LASIGE, Faculdade de Ciências, Universidade de Lisboa, Lisboa, Portugal; 3 Center of Technology and Systems (UNINOVA-CTS) and Associated Lab of Intelligent Systems (LASI), Universidade Nova de Lisboa, Lisboa, Caparica, Portugal; 4 Instituto Universitário de Lisboa (ISCTE-IUL), ISTAR, Lisboa, Portugal

**Keywords:** Deep learning, Neural networks, Recurrent neural networks (RNNs), Transformer models, Predictive applications, Accuracy in forecasting

## Abstract

Given the increasing need for accurate weather forecasts, the use of neural networks, especially transformer and recurrent neural networks (RNNs), has been highlighted for their ability to capture complex patterns in time series. This study examined 14 neural network models applied to forecast weather variables, evaluated using metrics such as median absolute error (MedianAbsE), mean absolute error (MeanAbsE), maximum absolute error (MaxAbsE), root mean squared percent error (RMSPE), and root mean square error (RMSE). Transformer-based models such as Informer, iTransformer, Former, and patch time series transformer (PatchTST) stood out for their accuracy in capturing long-term patterns, with Informer showing the best performance. In contrast, RNN models such as auto-temporal convolutional networks (TCN) and bidirectional TCN (BiTCN) were better suited to short-term forecasting, despite being more prone to significant errors. Using iTransformer it was possible to achieve a MedianAbsE of 1.21, MeanAbsE of 1.24, MaxAbsE of 2.86, RMSPE de 0.66, and RMSE de 1.43. This study demonstrates the potential of neural networks, especially transformers, to improve accuracy, providing a practical and theoretical basis for selecting the most suitable models for predictive applications.

## Introduction

As weather forecasting advances, it has been marked by the adoption of artificial intelligence (AI) models capable of capturing complex patterns in weather data, resulting in increasingly accurate forecasts (Hyndman, 2018). On the other hand, models such as GraphCast and Pangu-Weather exemplify this evolution, employing deep neural networks and sophisticated machine-learning techniques to predict weather conditions with high accuracy and speed. These AI-based innovations outperform traditional methods in terms of efficiency, offering forecasts of up to 10 days in just seconds (Lam et al., 2022; Bi et al., 2022). Consequently, neural network models have been widely used to increase the accuracy of weather time series forecasts. Architectures such as long short-term memory (LSTM) and transformers demonstrate a greater ability to detect seasonal patterns and extreme events more efficiently than traditional methods (Hittawe et al., 2024).

Accurate weather forecasting is vital across sectors such as agriculture (Ukhurebor et al., 2022), disaster preparedness (Rakhonde et al., 2024), and energy management (Meenal et al., 2022). In recent years, AI-driven methods, particularly deep learning approaches, have gained prominence for their ability to model complex, non-linear patterns in meteorological time series data. Foundational studies have explored the capabilities of recurrent neural networks (RNNs) like LSTM (Guillén-Navarro et al., 2019) and gated recurrent units (GRUs) (Jin et al., 2020) for sequential prediction tasks, while more recent work has demonstrated the superior long-range modeling power of transformer-based architectures.

Despite these advancements, a key limitation in the existing body of research is the lack of direct, head-to-head comparisons between different neural network architectures using real-world (Prendin et al., 2021), single-station weather data. Most studies either evaluate models in isolation, use synthetic or aggregated datasets, or vary the evaluation criteria, making it difficult to draw practical conclusions about model suitability in real-world forecasting applications.

This study addresses that gap by conducting a systematic comparison of 14 state-of-the-art neural network models, including both RNN and transformer variants, within a standardized framework using real weather data from a single station. By applying consistent evaluation metrics across models and focusing on both short- and long-term forecasting horizons, this work aims to provide actionable insights into model selection for operational forecasting tasks.

Chen et al. (2021) and Zhao, Xiong & Zhu (2024), show that these neural networks achieve significant results in climate forecasting, offering a predictive model that evolves with the inclusion of real-time data. These advances improve the quality of weather forecasts and contribute to a greater understanding and mitigation of the impacts of climate change (dos Santos et al., 2023). Thus, neural networks, including transformer models and RNNs, have emerged as effective technologies to address these challenges (Sherstinsky, 2020; Devlin, 2018; Liu, Bai & Wang, 2024).

Choosing the right approach can be a difficult task since in some cases filter applications can be interesting considering the high non-linearity of the signals (Moreno et al., 2024). In this context, many hybrid methods have been proposed to reduce noise in the signal, thus improving the predictive capacity of the models (Larcher et al., 2024). In addition, multi-criteria optimization techniques are applied to hypertuning the model to make the best possible use of the structure.

The transformer, introduced by Vaswani (2017), proposed an architecture based exclusively on attention mechanisms, eliminating the need for recurrent and convolutional layers. This enabled a significant increase in parallelization and reduced training time in language translation tasks while maintaining or even surpassing the accuracy of previous models (da Silva, Finardi & Stefenon, 2024). In this sense, unlike RNNs, which process inputs sequentially, the transformer uses global attention mechanisms to model dependencies at any position in the sequence. This feature makes the transformer more efficient in terms of computational complexity and especially suitable for long-range tasks such as machine translation and language modeling (Mandal, 2024; Tucudean et al., 2024). In short, models such as RNNs and transformers have been adopted to capture these dynamics, allowing for more robust and adaptive predictions. Recent research shows that these architectures not only capture seasonal patterns but also detect long-term trends that contribute to more accurate climate modeling (Lim & Zohren, 2021).

While RNNs process input sequentially, each hidden state ${h_{t}}$ depends on the previous state ${h_{t - 1}}$ and the current token, which allows the model to capture contextual information throughout the sequence. In contrast, the transformer uses an attention-based structure that eliminates recursion by treating all the tokens in the input simultaneously (Graves & Graves, 2012). Thus, transformer and RNNs adopt different strategies to capture dependencies in sequences: while the transformer exploits parallelization and distributed attention to process all input positions simultaneously, RNNs advance position by position, following a linear sequence. Each approach has particular benefits that may be better suited to specific contexts and types of sequential modeling tasks (Vaswani, 2017).

The NeuralForecast library is a platform available for Python (Olivares et al., 2022), which makes it possible to implement a wide range of temporal forecasting models based on neural networks, from traditional approaches, such as RNNs and multi-layer perceptron (MLP), to advanced architectures, such as transformers, neural basis expansion analysis for time series forecasting (NBEATS) (Oreshkin et al., 2021), neural hierarchical interpolation for time series forecasting (NHITS) (Challu et al., 2023), and temporal fusion transformer (TFT) (Nazir et al., 2023).

These models are widely applicable and can even be used for comparative analyses. With a unified interface, NeuralForecast makes it easy to integrate these models into forecasting workflows, meeting the needs of researchers and professionals who handle time series analysis with a high degree of adaptability. In addition, the library offers support for exogenous variables, allowing the inclusion of external factors that impact the time series, and has automatic hyperparameter adjustment features, optimizing the efficiency and accuracy of model configuration (Olivares et al., 2022).

Recent advances in deep learning have substantially improved time series forecasting by capturing complex spatiotemporal dependencies in diverse fields (Qi, Xu & Wang, 2025), with the TFT achieving state-of-the-art results in a range of domains (Sakib et al., 2024). For instance, Lam et al. (2023) developed GraphCast, an innovative machine learning framework that directly learns from decades of reanalysis data to produce skillful medium-range global weather forecasts at high resolution in less than a minute, demonstrating the promise of data-driven approaches in modeling highly non-linear dynamical systems.

In a related vein, Bi et al. (2022) introduced Pangu-Weather, a three-dimensional high-resolution model that integrates novel architectural strategies such as hierarchical temporal aggregation to achieve fast and accurate global weather forecasts while maintaining physical consistency across many weather variables.

Complementing these weather-focused studies, Wu (2023) compared the performance of several transformer-derived architectures, namely Transformer, Informer, Autoformer, and Non-Stationary Transformer, in forecasting financial market time series, thereby highlighting both the potential and limitations of modern neural network structures in domains characterized by non-stationarity and complex temporal patterns.

Despite the TFT demonstrated capabilities, there is a lack of research focused on leveraging a rigorously hypertuned TFT architecture for flow forecasting. This study addresses that gap by evaluating the performance of a finely optimized TFT model in the context of flow prediction. The model’s effectiveness is systematically compared against established deep learning baselines, specifically LSTM networks (Jiang et al., 2025), and TCN (Dudukcu et al., 2023). The evaluation includes a thorough hyperparameter optimization process, and the final results are benchmarked against a state-of-the-art forecasting framework (Kang et al., 2025).

While current models have shown encouraging performance in addressing the forecasting challenges, significant opportunities remain to enhance time series modeling architectures, particularly in effectively capturing nonlinear dynamics and long-range temporal dependencies. Lim et al. (2021) proposed the TFT, a transformer-based architecture, as a robust framework for time series forecasting. In a comparative study, Srivastava & Cano (2022) evaluated various deep learning architectures, including LSTM, gated recurrent units, RNN, and TFT, for the prediction levels. Their results demonstrated the superior performance of the TFT model, which consistently outperformed the other architectures, indicating its efficacy in time series forecasting.

Whereas transformers excel in handling long-range dependencies and parallelizing computations, features that are particularly beneficial when training on large datasets with complex patterns, RNNs possess inherent advantages in different scenarios. Due to their sequential nature and simpler structure, RNNs can be more effective when working with smaller datasets or tasks that rely on short-range dependencies, offering faster convergence and reduced computational overhead. A direct comparison highlighting these strengths and limitations would not only underscore the versatility of transformers but also clarify in which contexts RNNs might still be the preferred choice (Karita et al., 2019).

Besides the state-of-the-art techniques employed for time series forecasting, the use of hybrid models and noise attenuation techniques can be a solution to improve the forecasting of the signals evaluated in this work. Many advances have been proposed in the field of machine learning considering practical implementations based on data considering hybrid models (Shah et al., 2024).

Given the increasing need for accurate weather forecasts, the use of neural networks, especially transformers and RNNs, has been highlighted for their ability to capture complex patterns in time series. While many existing studies evaluate these models in isolation, few directly compare a broad set of neural network architectures on the same local weather station dataset. This study addresses that gap by examining 14 neural network models applied to forecast weather variables, all evaluated using consistent metrics such as median absolute error (MedianAbsE), mean absolute error (MeanAbsE), maximum absolute error (MaxAbsE), root mean squared percent error (RMSPE), and root mean square error (RMSE).

Accurate weather forecasting remains a critical challenge, especially at the local level where small-scale variations can significantly impact predictions. While neural networks have shown promise in capturing complex temporal dynamics, most existing studies evaluate these models in isolation or across varying datasets and conditions. Few studies conduct direct, head-to-head comparisons of multiple architectures using real-world, single-station weather data within a unified evaluation framework. This study addresses that gap by systematically comparing 14 neural network models for weather forecasting, and applying consistent evaluation metrics to ensure fair comparison.

In this context, the choice of the NeuralForecast library is based on its ability to integrate data in a simplified way, as well as offering advanced modeling options such as MLP, NBEATS, NHITS, and TFT (Olivares et al., 2022). This library facilitates the automatic adjustment of hyperparameters and the use of exogenous variables, contributing to the creation of models that more accurately reflect the variability of meteorological data. As a result, 14 models based on transformer architectures and RNNs were evaluated for predicting weather patterns, using the NeuralForecast library.

The experimental structure enabled a precise analysis of each model’s ability to identify seasonal patterns, variations, and long-term trends, which are essential aspects of climate data. In addition, by measuring the performance of these models, we sought a detailed assessment of their accuracy and efficiency, exploring the advantages offered by different architectures in climate modeling. The code for the 14 models developed for the analysis is available in a GitHub repository (dos Santos, 2025).

Following, we detail the methodology employed in this study, which includes a systematic review of the literature to identify the most effective methods in transformer models and RNNs applied to weather data forecasting. We present our approach to data collection and analysis, using the NeuralForecast library, which allows us to integrate real-time information to optimize our predictive models. We discuss the results obtained, comparing the effectiveness of different models in predicting extreme weather conditions. Finally, we explore the implications of these results for future research and practical applications in meteorology, to contribute to the advancement of climate forecasting technologies and improve the accuracy of weather forecasts.

## Methods

In this section, we present the methodological stages of this research, which focus on the identification and analysis of models based on transformers and RNNs applied to time series forecasting. The methodology was structured in four phases: systematic review, including the construction of the search string and the screening of relevant studies; collection and pre-processing of meteorological data from a local station; configuration, training, and cross-validation; and comparative evaluation of the predictive performance of the models based on metrics such as RMSE, RMSPE, MaxAbsE, MeanAbsE, and MedianAbsE (Stefenon et al., 2025).

### Search string construction and databases

To conduct the review on the application of transformer models and RNNs in weather data forecasting, with a focus on performance metrics, we used the following search string in the Google Scholar database (dos Santos et al., 2023) Google Scholar is a free-access platform that brings together various formats of academic publications and is currently one of the most comprehensive databases of scientific articles (Martín-Martín et al., 2021).

#### Selection process

The inclusion and exclusion criteria were articles that explored the use of transformers and RNNs exclusively focused on weather forecasting and included specific evaluation metrics. We excluded studies without a focus on weather forecasting or without the use of neural networks.

The screening of articles was carried out in three stages: In the first stage, we reviewed the titles to identify studies that explicitly mentioned neural networks, transformer or recurrent, applied to automatic weather station data. In the second stage, we analyzed the abstracts of the articles to assess their methodological relevance, verifying the description of performance metrics such as RMSE, RMSPE, MaxAbsE, MeanAbsE, and MedianAbsE. Finally, we read the selected articles in full to confirm their practical application in the context of weather forecasting.

### Local weather station data

The meteorological data used in this work was developed and collected in Brazil, in partnership with the Federal Institute of Paraná, located in Capanema, Paraná, Brazil. This collection covered real-time measurements over the period from January 1 to December 31, 2023, resulting in a total of 8,761 hourly records stored in CSV format (dos Santos, Beko & Leithardt, 2023). The partnership with the Federal Institute of Paraná lends greater credibility to the study, reinforcing the link with teaching and research institutions of excellence in Brazil. In addition, there is the prospect of expanding the scope of this work to include data analysis at an international level, increasing its relevance and global applicability.

Table 1 shows the meteorological variables collected by station WS-2080, organized into three columns: variable name, the unit of measurement, and data type. This table documents the main variables collected, serving as a basis for identifying patterns and exploring relationships between meteorological data and the environmental phenomena of interest, aligning directly with the objectives of the study.

**Table 1:** Layout of the variables of the local weather station analyzed.

Variable	Un. Measure	Type
Date	–	Date
Time	–	Time
Total precipitation	mm	Float
Atmospheric pressure	mB	Float
Max atmospheric pressure	mB	Float
Atmospheric pressure min	mB	Float
Global radiation	Kj/m^2^	Float
Air temperature (dry bulb)	°C	Float
Breakfast temp	°C	Float
Temp max	°C	Float
Min temp	°C	Float
Oven temp max	°C	Float
Water temp min	°C	Float
Rel. humidity max	%	Int
Humidity rel. min	%	Int
Relative air humidity	%	Int
Wind direction	Degrees	Int
Windbreak	m/s	Float
Wind speed	m/s	Float

For further analysis, the dataset used in this article is available at dos Santos (2025). To prepare the data used, a pre-processing stage was handled to structure the time series in a way that was compatible with the requirements of the learning algorithms. The process began by reading the meteorological data file, stored in CSV format.

The date (Date) and time (Time) columns were combined and converted to datetime format, giving rise to the ds column, which represents the timestamp of each observation. Records that could not be correctly converted to datetime format were removed to ensure the temporal consistency of the series. The variable of interest selected for forecasting was the air temperature measured by the dry bulb (°C), extracted from the corresponding column and renamed $y$, according to the standard nomenclature used in time series libraries such as NeuralForecast and Prophet. To enable the set to be used in contexts with multiple series, a $unique {-}id$ column was added with a fixed value $serie {-} 1$, allowing the series to be uniquely identified within multivariate or multi-ID structures.

Finally, records with missing values in the $y$ variable were eliminated, and the set was sorted in ascending order based on the time column (ds), with the index restarted. This procedure guarantees the integrity and chronological ordering required for proper modeling of the time series. The records of the database considered represent a time series that is analyzed in this article, these signals present non-linearities due to intrinsic characteristics of the measurement recorded on site. These characteristics make prediction a challenge, and sophisticated measurements are required to achieve acceptable performance. The signal considered has all the samples recorded, with no missing data, sparse data, or considerable outliers.

In this comparative study of machine learning model architectures (RNNs and transformers) applied to weather forecasting, we chose to use air temperature (dry bulb) as the only target variable. This methodological decision was motivated by the need to prioritize the effectiveness of the analysis, to the detriment of including other strongly correlated variables. The univariate approach significantly simplified the comparison between the different models, allowing for a more direct and objective interpretation of the results. The inclusion of additional variables would increase the complexity of the analysis, making it difficult to accurately identify the strengths and weaknesses of each architecture, as some architectures do not support co-variables.

Furthermore, the choice also took into account practical limitations related to available computing resources and processing time. Multivariate models require significantly more computing power, as well as longer training times. The limited data available, from a single local weather station, influenced this decision. For more complex multivariate models, more robust data sets are essential to guarantee the quality of the training and avoid the problem of overfitting. Thus, the use of a single target variable helped mitigate this risk, ensuring more complete and generalizable results under the conditions proposed in this study.

### Data pre-processing

The data from the weather station went through a preparation and pre-processing process. The steps included formatting the temporal information, defining the target variable, and dealing with missing or inconsistent data. Once the target variable had been defined, it was prepared for use in the models, following methodologies such as those described by Bilgin et al. (2021), which emphasize the importance of pre-processing in time series forecasts to ensure accuracy and consistency in the results.

To deal with missing values in the data, we used linear interpolation for temporal variables, such as “Air Temperature”, and imputation by the mean for variables such as “Relative Humidity”. For cases with high inconsistency, the lines were removed after analyzing the impact on the time series. Timestamp Formatting results from the date and time columns being combined into a single ds column, ensuring an uninterrupted time sequence. The y column, representing air temperature (dry bulb), was selected as the target variable for the forecasts.

#### Target variable

The choice of air temperature (dry bulb) as the target variable is based on its relevance as a central indicator of meteorological conditions, directly impacting processes essential to the study, such as evapotranspiration and soil moisture dynamics, both important for understanding and predicting climatic variations. In addition, its stability over time and the wide availability of historical data reinforce its suitability for predictive analysis in time series, allowing for greater precision and representativeness of environmental conditions in models developed in this work (Fister et al., 2022).

#### Variation of the target variable over time

**Figure 1:**
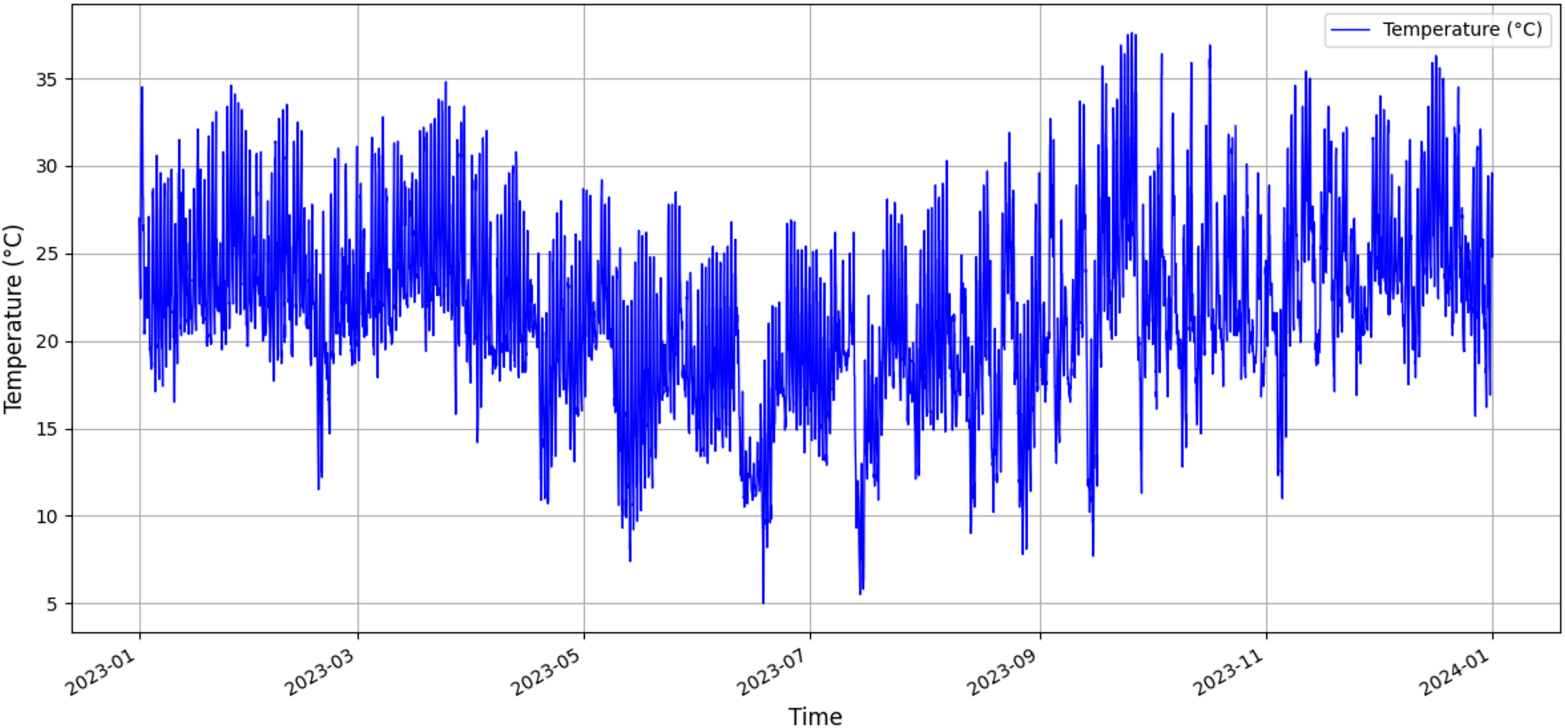
Variation in air temperature (dry bulb) over time.

The graph presented in Fig. 1 was designed to show the variation in air temperature (dry bulb) over time, from January 2023 to April 2024. Temperatures were recorded approximately between 10 °C and 35 °C, revealing significant fluctuations, with the highest temperatures during September to December and the lowest during the periods of June and July. This analysis of temperature variations allows for a detailed assessment of climate trends and can be correlated with other meteorological data sets.

### Correlations

**Figure 2:**
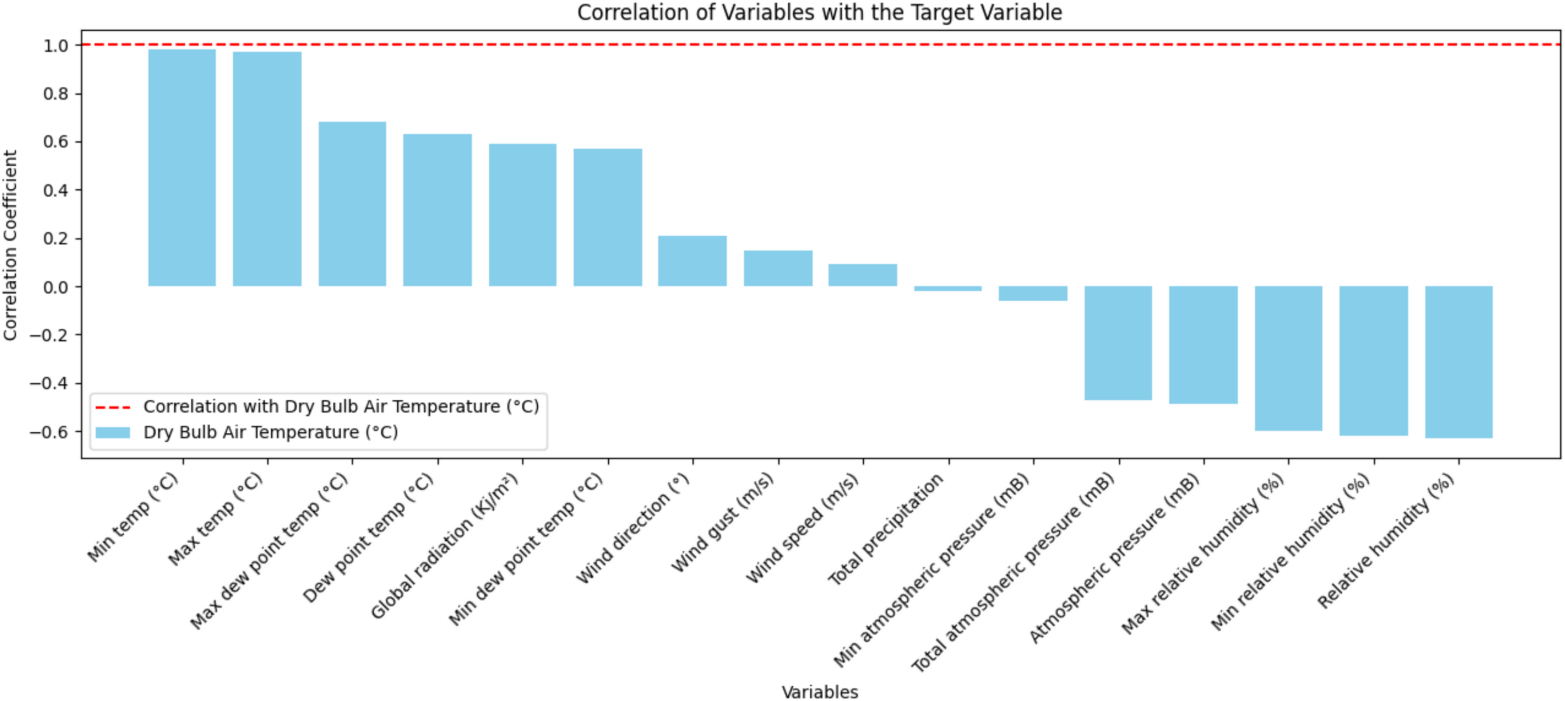
Correlation between the target variable and the other meteorological variables.

The plot shown in Fig. 2 illustrates the correlations between the target variable, dry bulb air temperature (°C), and the other meteorological variables, showing how these variables are linearly related. It can be seen that the variables temp min (°C) and temp max (°C) stand out as the most positively correlated, indicating that they both behave similarly to the target variable. This relationship shows that these variables are directly linked to the thermal patterns recorded and could be considered key predictors in future analyses.

On the other hand, variables such as relative humidity (%) and atmospheric pressure show moderate negative correlations, suggesting that these quantities are inversely associated with dry bulb temperature. This reflects important climatic interactions, indicating that as humidity or atmospheric pressure increases, temperature tends to decrease. Variables such as global radiation (Kj/m^2^) show a moderate positive correlation, pointing to a relevant but not predominant influence of solar radiation on temperature variation.

Some variables, such as wind speed (m/s), show a correlation close to zero, which implies little or no linear relationship with dry bulb temperature, showing that these variables may be less relevant for predictive modeling. This analysis makes it possible to understand the interactions between the meteorological variables and the target variable, guiding the selection of more significant variables and reinforcing the understanding of the underlying climatic processes. In this way, the graph establishes a clear overview of the relationships and is essential for guiding subsequent modeling and analysis steps.

### Model configuration

The transformer and RNNs models were configured to evaluate their performance in forecasting meteorological time series, with a focus on automatically selecting the best hyperparameters and adapting them to the validation set. Studies such as Nguyen et al. (2023) highlight the effectiveness of these architectures when tackling complex temporal forecasting problems, demonstrating performance improvements when integrating exogenous variables, and configuring the models for univariate or multivariate forecasts. Table 2 presents a summary of the characteristics of each model used, including architecture, univariate or multivariate configuration, type of forecast, and support for exogenous variables (Olivares et al., 2022).

**Table 2:** Summary of characteristics of the transformer-based and RNN-based models used in forecasting.

Model	Architecture	Univariate/Multivariate	Forecast type	Exogenous
TFT	Transformer	Univariate	Direct	F/H/S
VanillaTransformer	Transformer	Univariate	Direct	F
Informer	Transformer	Multivariate	Direct	F
Former	Transformer	Univariate	Direct	F
FEDformer	Transformer	Univariate	Direct	F
PatchTST	Transformer	Univariate	Direct	–
iTransformer	Transformer	Multivariate	Direct	–
RNN	RNN	Univariate	Recursive	F/H/S
LSTM	RNN	Univariate	Recursive	F/H/S
GRU	RNN	Univariate	Recursive	F/H/S
TCN	RNN	Univariate	Recursive	F/H/S
DeepAR	RNN	Univariate	Recursive	F/S
DilatedRNN	RNN	Univariate	Recursive	F/H/S
BiTCN	RNN	Univariate	Direct	F/H/S

### Structural parameters

When configuring machine learning models for weather time series forecasting, it was essential to adjust parameters such as the number of layers, batch size, learning rate, and number of neurons in each layer to maximize predictive accuracy as illustrated in Table 3. These adjustments are essential to capture the complexities of meteorological data and improve the model’s performance (Gomes & Ludermir, 2021).

**Table 3:** Table of hyperparameters values used in each model.

Model	Parameters
RNN	h, input_size, inference_input_size, loss=MQLoss, scaler_type, encoder_n_layers, encoder_hidden_size, context_size, decoder_hidden_size, decoder_layers, max_steps, full_horizon, model_name
LSTM	h, input_size, loss=DistributionLoss, scaler_type, encoder_n_layers, encoder_hidden_size, context_size, decoder_hidden_size, decoder_layers, max_steps, full_horizon, model_name
GRU	h, input_size, loss=DistributionLoss, scaler_type, encoder_n_layers, encoder_hidden_size, context_size, decoder_hidden_size, decoder_layers, max_steps, full_horizon, model_name
TCN	h, input_size, loss=GMM, learning_rate=5e−4, kernel_size=2, dilations=[1, 2, 4, 8, 16], encoder_hidden_size, context_size, decoder_hidden_size, decoder_layers, scaler_type, max_steps, full_horizon, model_name
DeepAR	h, input_size, lstm_n_layers=3, trajectory_samples=100, loss=DistributionLoss, learning_rate=0.005, max_steps, val_check_steps, early_stop_patience_steps, scaler_type=standard, full_horizon, model_name
DilatedRNN	h, input_size, loss=DistributionLoss, scaler_type=robust, encoder_hidden_size, max_steps, full_horizon, model_name
BiTCN	h, input_size=24, loss=GMM, max_steps=100, scaler_type=standard, full_horizon, model_name
TFT	h, input_size=tune.choice([horizon]), hidden_size=tune.choice([8, 32]), n_head=tune.choice([2, 8]), learning_rate=tune.loguniform(1e−4, 1e−1), scaler_type=tune.choice([robust, standard]), max_steps=tune.choice([500, 1,000]), windows_batch_size=tune.choice([8, 32]), check_val_every_n_epoch=tune.choice([100]), random_seed=tune.randint(1, 20), num_samples=10, freq=’H’, save_dataset=True, overwrite=True, full_horizon, model_name
VanillaTransformer	h, input_size=horizon, hidden_size=16, conv_hidden_size=32, n_head=2, loss=MAE, scaler_type=robust, learning_rate=1e−3, max_steps=500, full_horizon, model_name
Informer	h, input_size=horizon, hidden_size=16, conv_hidden_size=32, n_head=2, learning_rate=1e−3, scaler_type=robust, max_steps=500, full_horizon, model_name
Former	h, input_size=horizon, hidden_size=16, conv_hidden_size=32, n_head=2, learning_rate=1e−3, scaler_type=robust, max_steps=500, full_horizon, model_name
FEDformer	h, input_size=24, modes=64, hidden_size=64, conv_hidden_size=128, n_head=8, learning_rate=1e−3, scaler_type=robust, max_steps=500, batch_size=2, windows_batch_size=32, val_check_steps=50, early_stop_patience_steps=2, full_horizon, model_name
PatchTST	h, input_size=104, patch_len=24, stride=24, revin=False, hidden_size=16, n_heads=4, scaler_type=robust, learning_rate=1e−3, max_steps=500, val_check_steps=50, early_stop_patience_steps=2, full_horizon, model_name
iTransformer	h, input_size=24, n_series=2, hidden_size=128, n_heads=2, e_layers=2, d_layers=1, d_ff=4, factor=1, dropout=0.1, use_norm=True, loss=MSE, valid_loss=MAE, early_stop_patience_steps=3, batch_size=32, full_horizon, model_name

### Training and cross-validation

The process of training (Santos, Beko & Leithardt, 2023) and cross-validating the models was carried out considering the model initialization, training, and cross-validation, and saving the results. Each model was configured with the forecast variables (ds, y, and unique_id) to ensure the integrity of the input data. During training, cross-validation was used on *k*-folds, dividing the input data into *k* subsets of equivalent size.

In each iteration, one subset was reserved for validation, while the others were used for training. The *early stopping* technique was applied in each iteration to avoid excessive training and speed up the process. After the end of each iteration, the trained models were saved in specific directories (checkpoints), and the validation results were stored for comparative analysis.

To respect the time domain, cross-validation was handled using a sliding window, in which future predictions take into account past observations, thus ensuring the correct application of the prediction. To avoid overfitting the model, the early stop criterion takes into account a comparison between the loss function of training and validation, when the model starts overfitting, where there is no further improvement in validation, the early stop is used.

### Multi-step forecasting

After training, the models performed multi-step forecasts to assess their long-range capacity (Moreno et al., 2024). To ensure accurate forecasts over a long horizon, it was divided into blocks (*batches*), allowing successive forecasts to be made from previous forecasts, as highlighted by Manawadu et al. (2022). Two forecasting methods were used, recursive forecasting and direct forecasting. In recursive forecasting, each forecast was performed one step at a time, with the previous forecast used as input for the next step. This approach has been widely used in time series tasks and allows flexibility in extending the forecast horizon.

Models that support direct forecasting generated all horizon steps at once, reducing the effect of error accumulation. This technique was especially effective in models such as transformers, due to its ability to model long-term dependencies (Nguyen et al., 2023). The forecasts generated were concatenated with the training data to simulate a scenario of sequential and continuous forecasts, to replicate a practical application situation.

### Performance evaluation

The performance of the models was evaluated using RMSE, RMSPE, MaxAbsE, MeanAbsE, and MedianAbsE. The RMSE is used to measure the magnitude of squared errors, suitable for capturing significant errors. This metric is widely used in time series forecasting (Ribeiro et al., 2024; Yamasaki et al., 2024). RMSPE assesses the relative accuracy of the models over the time series. MaxAbsE records the maximum absolute error in each forecast, useful for identifying atypical errors. MeanAbsE represents the mean absolute error, providing an overview of the model’s performance over the series. MedianAbsE measures the median absolute error, a robust metric against outliers.

The equation to calculate these measures are given by:

(1) $${\rm  RMSE} = \sqrt {{1 \over n}\sum\limits_{i = 1}^n {{{({y_{i}} - {{\hat y}_i})}^2}} }$$

(2) $${\rm RMSPE} = \sqrt {{1 \over n}\sum\limits_{i = 1}^n {{{\left( {{{{y_{i}} - {{\hat y}_i}} \over {{y_{i}}}}} \right)}^2}} } \times 100$$

(3) $${\rm MaxAbsE} = {\max _{i}}\left| {{y_{i}} - {{\hat y}_i}} \right|$$

(4) $${\rm MeanAbsE} = {1 \over n}\sum\limits_{i = 1}^n {\left| {{y_{i}} - {{\hat y}_i}} \right|}$$

(5) $${\rm MedianAbsE} = {\mathrm{median}} \left( {\left| {{y_{1}} - {{\hat y}_1}} \right|, \ldots ,\left| {{y_{n}} - {{\hat y}_n}} \right|} \right)$$

where ${y_{i}}$ is the actual (true or observed) value for the ${i^{th}}$ data point, ${\hat y_{i}}$ is the predicted (or estimated) value for the ${i^{th}}$ data point, and $n$ is the total number of data points (Klaar et al., 2023).

These metrics were used based on the lightning_logs directory and are available in the repository of dos Santos (2025), enabling a detailed and transparent comparative analysis between the models. The application of these robust evaluation metrics ensured a comprehensive understanding of the predictive performance of each model, helping to identify the most suitable and accurate approaches for forecasting weather time series.

#### Hardware

The processing times reported in this study were measured on a specific hardware configuration, which includes 8.00 GB of RAM and an Intel Core i5-10210U processor with a base frequency of 1.60 GHz and the ability to accelerate to 2.11 GHz under high load conditions.

#### Comparison of processing times and normalization methods

The processing times and normalization methods evaluated in this study were fundamental to the comparative analysis between the 14 forecasting models, as shown in Table 4. The methodological approach adopted was based on the standardized execution of each model in a controlled hardware environment. Processing times were measured to identify the computational efficiency of each model, while the normalization methods applied, such as robust and standard, made it possible to assess the adaptability of the models to different data distributions and scales.

**Table 4:** Comparison of models with processing times and normalization applied.

Models	Time (min)	Normalization “scaler_type”:
TFT	18.49	robust, standard
RNN	17.08	robust
LSTM	42.49	robust
GRU	59.15	robust
TCN	2.34	robust
DeepAR	6.90	standard
DilatedRNN	3.40	robust
BiTCN	0.83	standard
VanillaTransformer	5.89	robust
Informer	6.26	robust
Former	8.98	robust
FEDformer	2.87	robust
PatchTST	2.03	robust
iTransformer	0.29	robust

The results showed that models such as BiTCN and iTransformer, with processing times of less than a minute, are highly efficient for scenarios that require speed. On the other hand, more robust models, such as LSTM and GRU, showed higher times, suggesting greater computational complexity, but potentially offering advantages in terms of accuracy under specific conditions. Finally, this analysis shows that a model’s choice should be based not only on processing time but also on the suitability of the normalization method and the model’s ability to meet the requirements of the problem in question.

The limitation of using predefined models is that tuning is required for the specific application, and predefined models for general applications may not be the best alternative when the task has other characteristics, such as time series signals with greater noise intensity. This makes it necessary to fine-tune the model to meet the necessary characteristics considering the signal being evaluated.

The analysis developed in this study was handled specifically with the data considered in this project, however, the models show promise for handling any chaotic time series. Future work can be carried out aiming to compare the methods evaluated in this study in different scenarios. In the next section, the results and comparison between the models evaluated are presented, highlighting those with the best performance and pointing out the most promising architectures for temporal forecasting applications of climate data to a local automatic weather station.

The results found in this study show that the models used meet prediction expectations satisfactorily. Considering the non-linearities of the signals analyzed, the same models can be applied to other data sets, as they are generalizable models. To enable future analyses and applications of the models used in this work in other fields, the data set and algorithms used are available at dos Santos (2025).

## Results

The focus of the analysis of the results is on the comparative evaluation of the performance of different models such as transformers and RNNs applied to forecasting meteorological data. Using five error metrics. These metrics made it possible to identify the most efficient model architectures in terms of error minimization and predictive stability, providing a basis for understanding the suitability of each model to the challenges of forecasting applied to weather station data.

**Figure 3:**
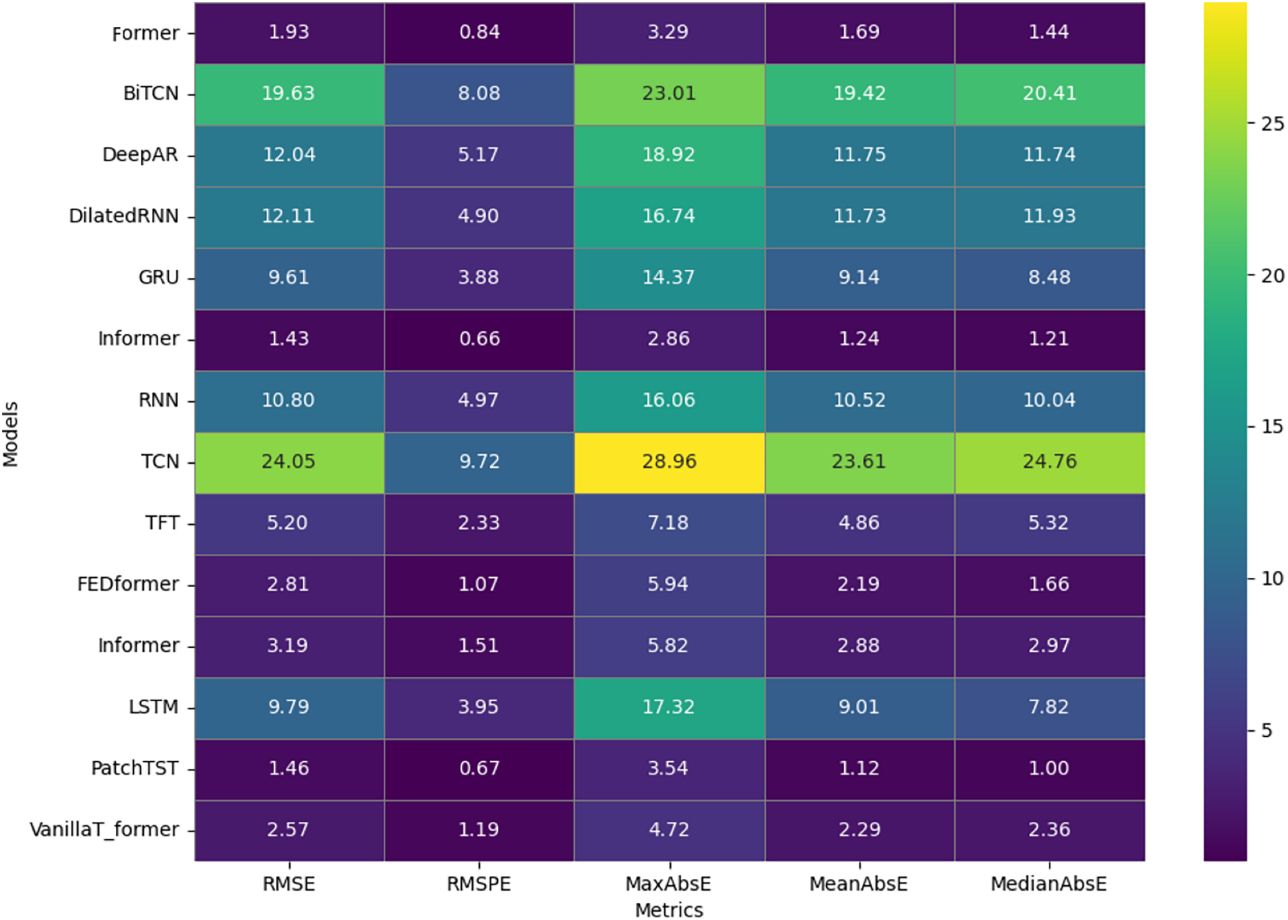
Heat map showing the performance of 14 models on five applied metrics.

Figure 3 shows a heat map detailing the results obtained when evaluating the performance of the 14 models based on the error metrics: RMSE, RMSPE, MaxAbsE, MeanAbsE, and MedianAbsE. This heat map uses a gradient of colors from green to purple, indicating a performance range of low (green) and high (purple) for each metric. The intensity of the color in each cell highlights the magnitude of the error, providing a quick visualization of which models perform better or worse than the different models tested.

The PatchTST model stands out for its low values in several metrics, such as RMSE (1.46) and MedianAbsE (1.00), which are represented by darker-colored cells, indicating superior performance. Similarly, the former model also performs with an RMSE of 1.93 and an RMSPE of 0.84, evidenced by dark shades that underline its high accuracy. In contrast, the BiTCN and TCN models show lighter colors to indicate the highest RMSE values, 19.63 and 24.05, respectively, suggesting significant limitations in their ability to predict complex temporal patterns compared to other models.

The TFT and Informer models, with RMSE of 5.20 and 3.19, respectively, are represented by moderate shades of color, positioning them as balanced alternatives that offer a compromise between accuracy and computational complexity. Finally, the VanillaTransformer and FEDformer models, with RMSEs of 2.57 and 2.81, appear in intermediate colors on the map, highlighting their effectiveness as good options due to the balance between accuracy and overall performance. Although all models based on transformer and RNN demonstrate predictive capacity, transformers such as PatchTST and Former stand out significantly. The superior accuracy of these models is evidenced by the darker colors, which indicate lower errors in the evaluation metrics.

### Evaluation of model metrics

**Figure 4:**
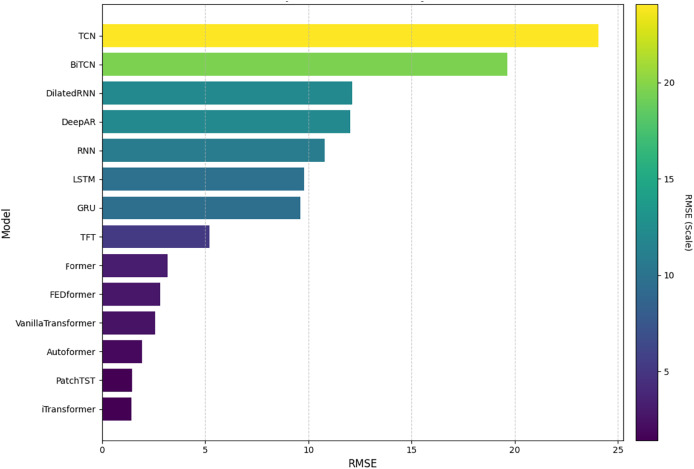
Comparison of model performance in terms of RMSE.

**Figure 5:**
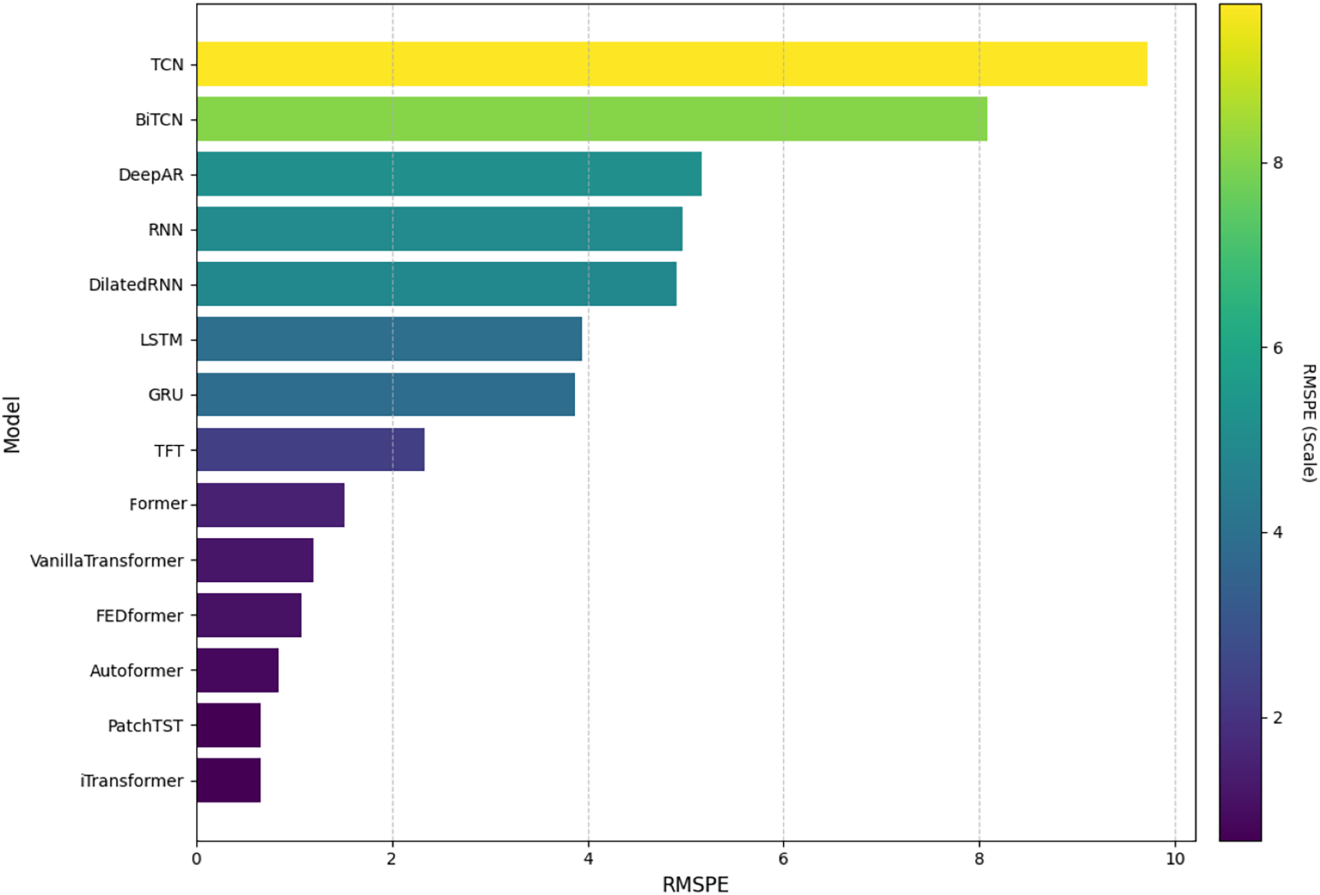
Comparison of model performance in terms of RMSPE.

**Figure 6:**
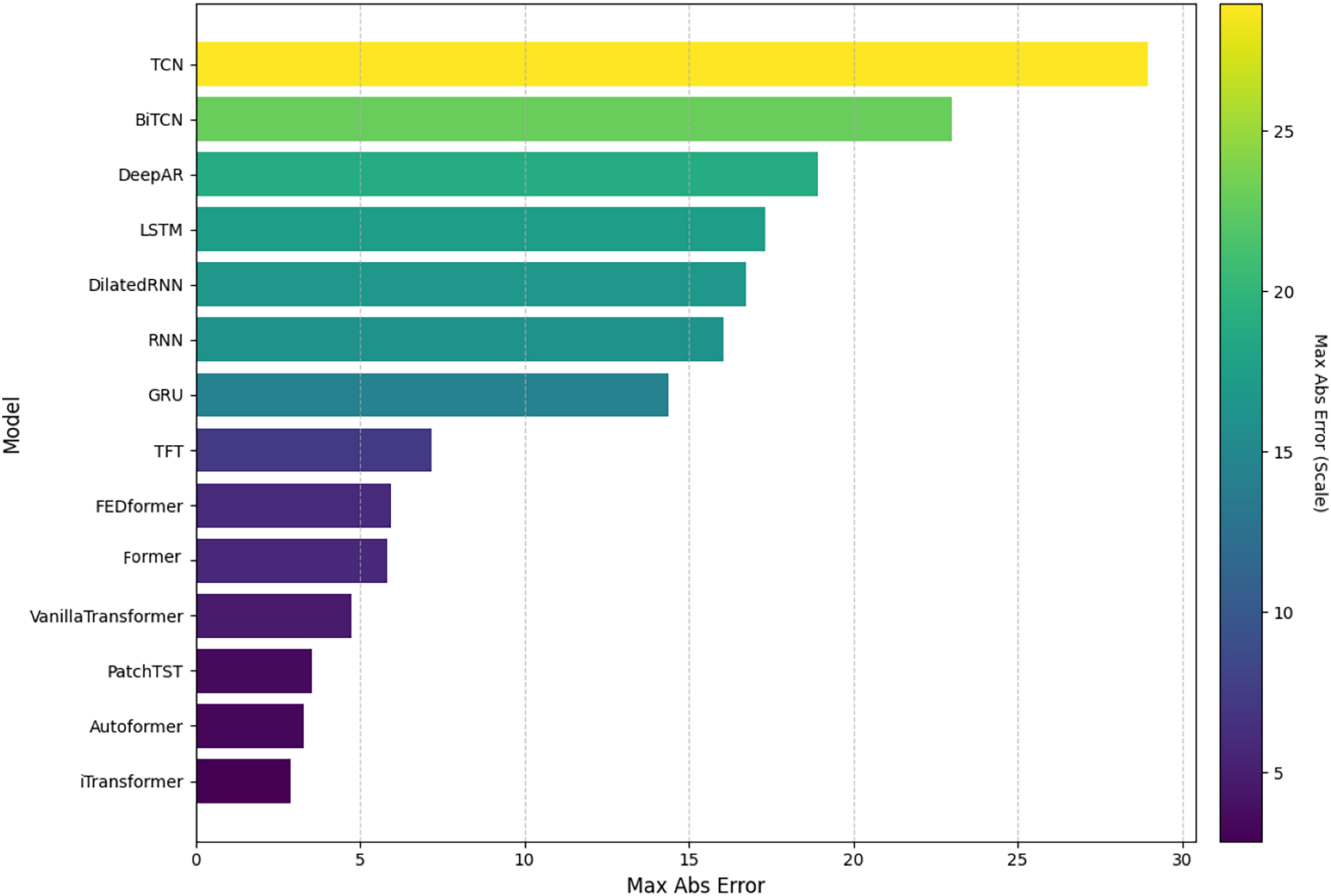
Comparison of model performance in terms of MaxAbsE.

**Figure 7:**
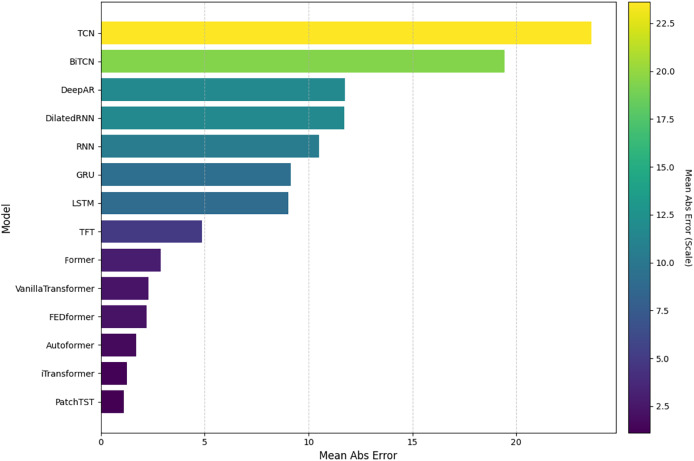
Comparison of model performance in terms of MeanAbsE.

**Figure 8:**
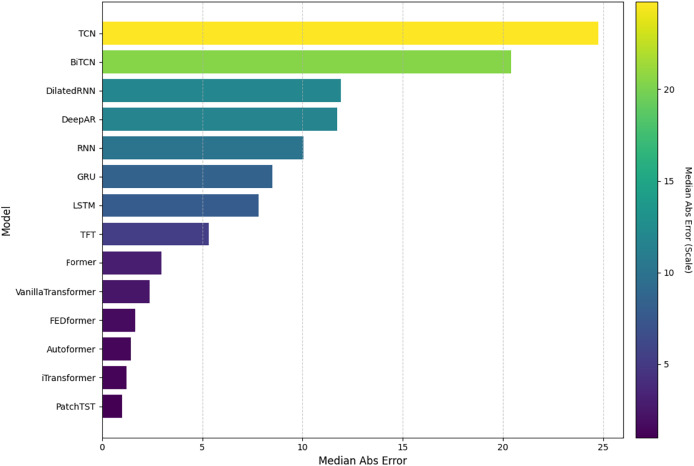
Comparison of model performance in terms of MedianAbsE.

In this analysis, we will present the individual results of the 14 models evaluated under the five metrics. Each metric provides a unique perspective on the effectiveness of each model in capturing and predicting complexities in weather time series. Each subsection details the models’ performance on a specific metric, starting with RMSE. The embedded Figs. 4, 5, 6, 7, and 8 provide a clear visual representation of these comparisons, highlighting both the models and the performance of those with their limitations.

#### Root mean squared error

Figure 4 shows a comparison of the models evaluated based on the RMSE, sorted in ascending order. It can be seen that the iTransformer model obtained the lowest RMSE value, showing high accuracy and forecasting capacity in time series. The PatchTST and Former models also performed well. On the other hand, the TCN and BiTCN models had the highest RMSE values, suggesting limitations in capturing complex patterns. Intermediate models, such as VanillaTransformer and TFT, showed reasonable results, falling between the extremes.

#### Root mean squared percentage error

Figure 5 shows a comparison of the models evaluated based on the RMSPE metric, also sorted in ascending order. The iTransformer model showed the lowest RMSPE value, standing out for its high percentage accuracy. The PatchTST and former models also stand out, reinforcing the effectiveness of these transformer-based architectures. However, the TCN and BiTCN models had the highest RMSPE values, indicating greater difficulty in dealing with percentage variations in the data. Other models, such as TFT and Informer, among others, offered intermediate performance.

#### Maximum absolute error

Figure 6 shows the comparison of the models based on the MaxAbsE. The iTransformer model obtained the lowest value, demonstrating superior control over extreme errors. The PatchTST and Former models also performed well in this respect. The highest absolute maximum error values were recorded for the TCN and BiTCN models, indicating greater sensitivity to spikes in the data. Models such as LSTM and TFT, among others, showed intermediate results.

#### Mean absolute error

Figure 7 shows the results concerning MeanAbsE. In this case, PatchTST stood out as the most accurate model, with the lowest mean absolute error. The iTransformer and Former models also performed well, with close values. On the other hand, the TCN and BiTCN models recorded the highest values, suggesting less stability in the forecasts. Intermediate models such as VanillaTransformer and Informer, among others, showed reasonable accuracy.

#### Median absolute error

Figure 8 illustrates the results for the MedianAbsE. The lowest value was obtained by the PatchTST model, followed by the iTransformer and Former models, showing consistency in their predictions. On the other hand, the TCN and BiTCN models had the highest values, indicating greater variability in their median results. Intermediate models include TFT and Informer, among others which show more balanced performance.

Based on the results presented, it was possible to identify that the 14 models evaluated exhibit varied performance when dealing with meteorological time series, with a consistent emphasis on transformer-based architectures, such as iTransformer, PatchTST, and Former. Despite this, it is important to emphasize that the performance of these models is intrinsically linked to the specific parameters and conditions used. Changes, such as adjustments to hyperparameters, variable selection, or data pre-processing, can have a significant impact on the results observed.

Although the TCN and BiTCN models showed greater limitations in the metrics evaluated, these observations do not rule out the possibility that improvements in their parameterizations could alter their predictive capabilities. Similarly, intermediate performance models such as TFT and Informer offer opportunities for further optimization, depending on the application context and specific objectives.

It is therefore concluded that the selection of a model for practical application should take into account not only the results obtained in this analysis but also the flexibility to explore and adjust configurations that meet the needs of the problem in question. This iterative approach was essential to ensure initial understanding while maintaining a balance between accuracy and computational performance.

## Discussion

The weather time series forecasting models presented in this analysis vary in terms of architecture, computational complexity, and suitability for different forecast horizons and frequencies. Each model offers specific benefits that can be taken advantage of depending on the application context, such as short, medium, or long-term forecasts, and the availability of historical and future data.

**Figure 9:**
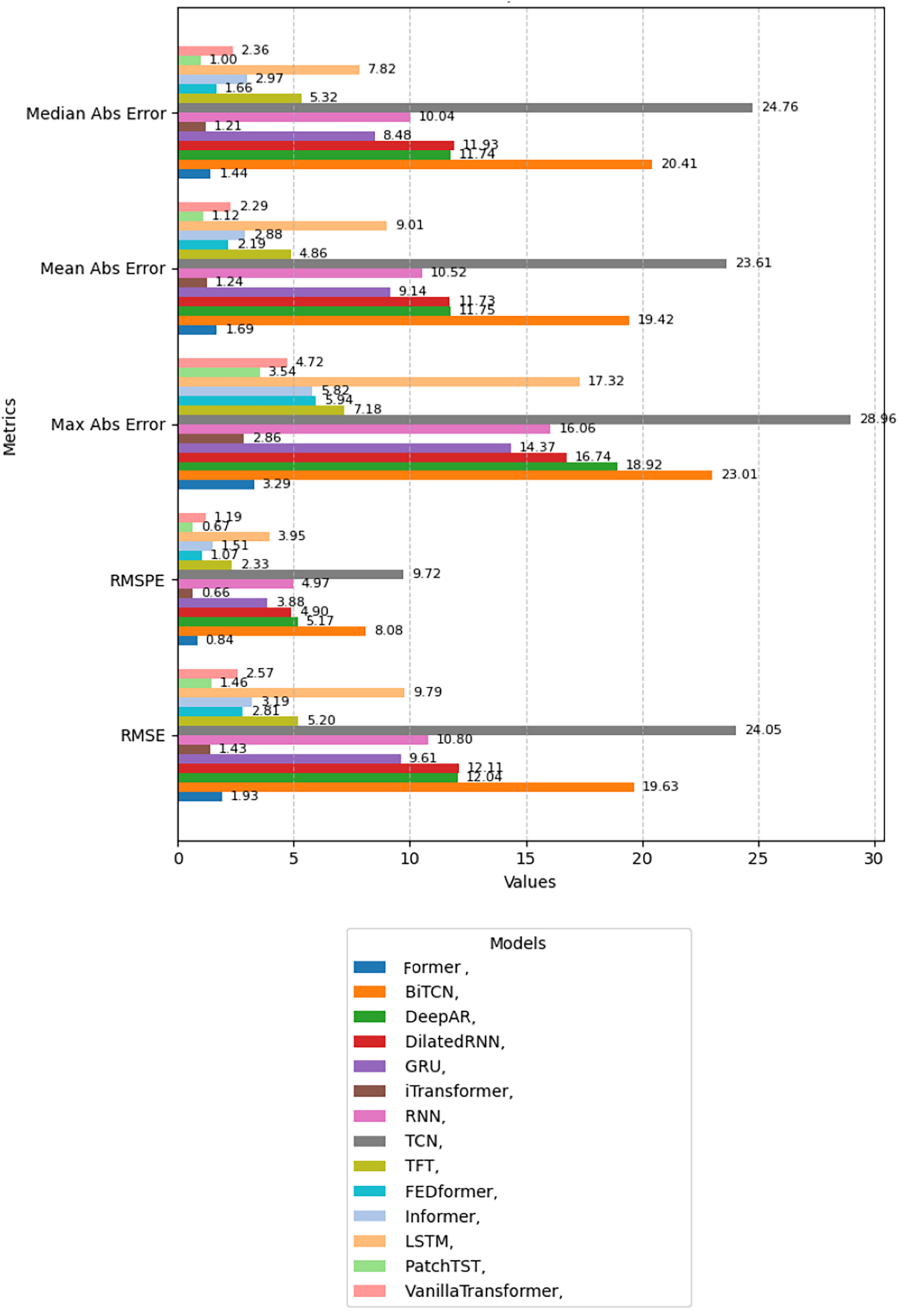
Performance comparison between different neural network models applied to forecasting meteorological variables.

The results presented in Fig. 9 provide a detailed analysis of the relative performance of the different models analyzed, by metrics and considered from a global point of view. These metrics were key to understanding different aspects of the models’ performance, such as overall accuracy, control of error peaks, and forecast stability. The diversity in the results highlights the importance of evaluating the models from multiple perspectives to select the most appropriate approach to the specific task.

Among the models evaluated, transformer-based architectures such as Former, PatchTST, and iTransformer showed the best performance. These models showed low variability in metrics such as RMSE and RMSPE, reflecting their high capacity to capture complex patterns in time series. In addition, the lower MaxAbsE values show these models’ superior control over extreme errors, an important aspect in applications that require high forecast accuracy and reliability.

On the other hand, models based on temporal convolutions, such as TCN and BiTCN, faced greater difficulties in practically all the metrics evaluated. Although efficient at capturing local patterns, these architectures showed limitations in dealing with more dynamic time series and long-term dependencies, resulting in higher absolute and percentage errors. This reinforces the importance of considering the characteristics of the data when selecting a model, especially in scenarios with high temporal complexity.

The intermediate performance models, such as TFT, VanillaTransformer, and Informer, showed moderate results, placing them between the extremes. These models showed balance in the metrics evaluated, making them suitable for applications where multiple factors influence the results. This makes them a viable option for less critical scenarios, where there is flexibility to explore parameters and adjust them according to the needs of the problem.

The analysis shows that architectural choices directly impact model performance, especially concerning metrics that assess accuracy and stability. Model selection must consider both the application’s objectives and the metrics that most closely match the demands of the usage scenario. In addition, the possibility of adjusting parameters and exploring different configurations was necessary to ensure that the chosen model could meet the expectations and specific characteristics of the data analyzed.

**Figure 10:**
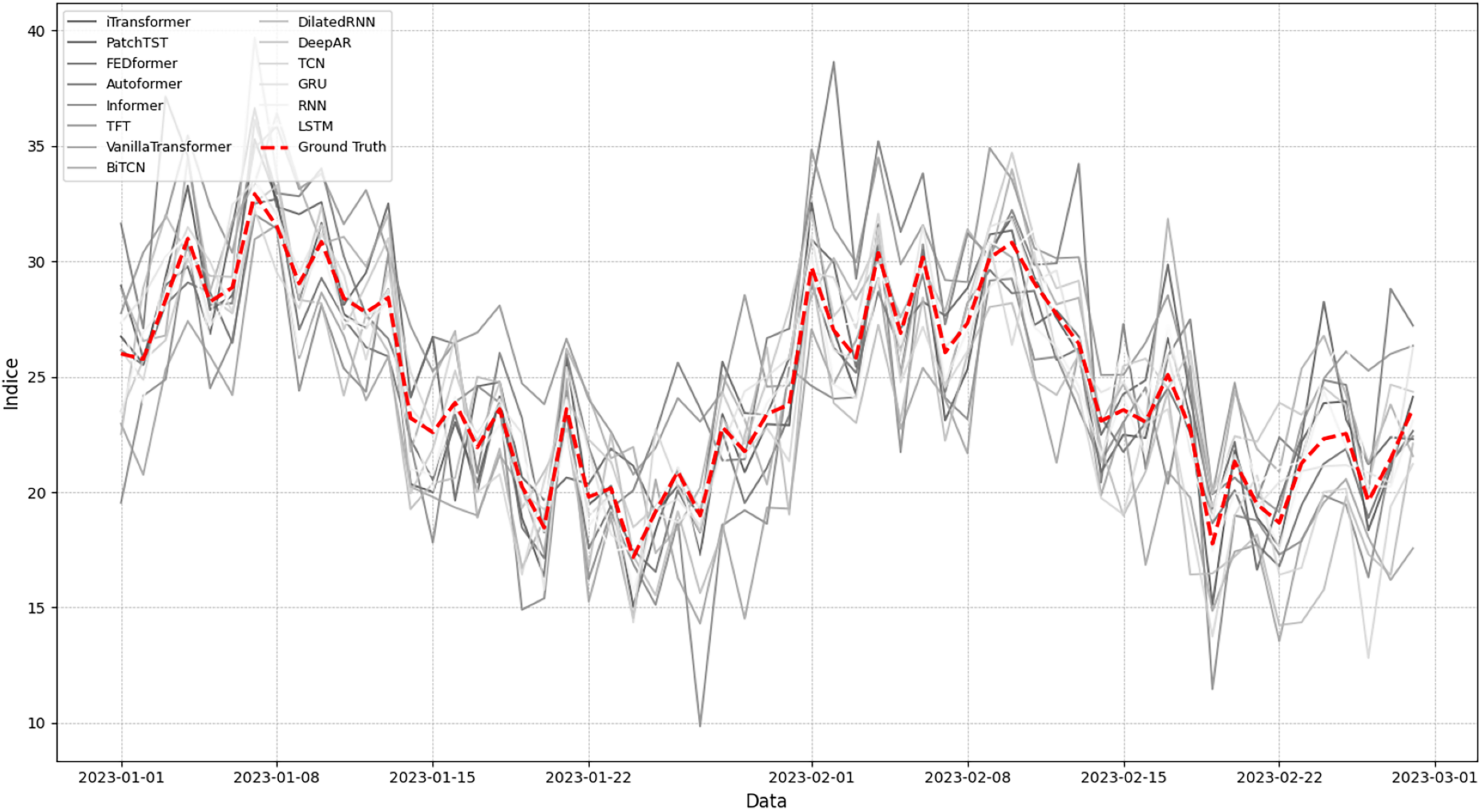
Comparison of the trends of predictive models against the ground truth.

The plot shown in Fig. 10 allows for a detailed analysis of the behavior of predictive models in relation to the ground truth over the time series. Models such as iTransformer and PatchTST are observed to exhibit greater stability, with smooth trajectories that follow the general trends of the data. This stability is essential in scenarios where precision and consistency are crucial for strategic decision-making. These models, based on transformer architectures, demonstrate a superior ability to capture long-term patterns, a highly relevant aspect in meteorological forecasts.

However, models such as BiTCN and TCN show greater sensitivity to rapid variations, resulting in more pronounced oscillations. This behavior can be a disadvantage in scenarios that require more stable forecasts, but it can also be advantageous in applications that require quick responses to abrupt changes, such as warning systems or anomaly detection. This reflects the intrinsic limitations of architectures based on temporal convolutions, which, although effective for local patterns, face challenges in capturing global trends.

The variation among models highlights the influence of architectures and parameterizations on predictive performance. Models such as LSTM and VanillaTransformer exhibit distinct patterns compared to more advanced approaches, indicating that the complexity of the architecture directly impacts its generalization and accuracy capabilities. This diversity underscores the importance of a careful analysis of the specific needs of each problem before selecting a model, considering the characteristics of the data and the prediction objectives.

Figure 10 also reinforces the need to evaluate models based on multiple criteria and contexts, as different applications may prioritize attributes such as stability, reactivity, or long-term accuracy. This analysis enables the identification of both the limitations and the potential applications of each model, promoting a more strategic use of time-series forecasting tools.

The choice of the ideal model should consider a combination of factors, including data characteristics, model architecture, and the specific demands of the application context (dos Santos, 2025).

**Figure 11:**
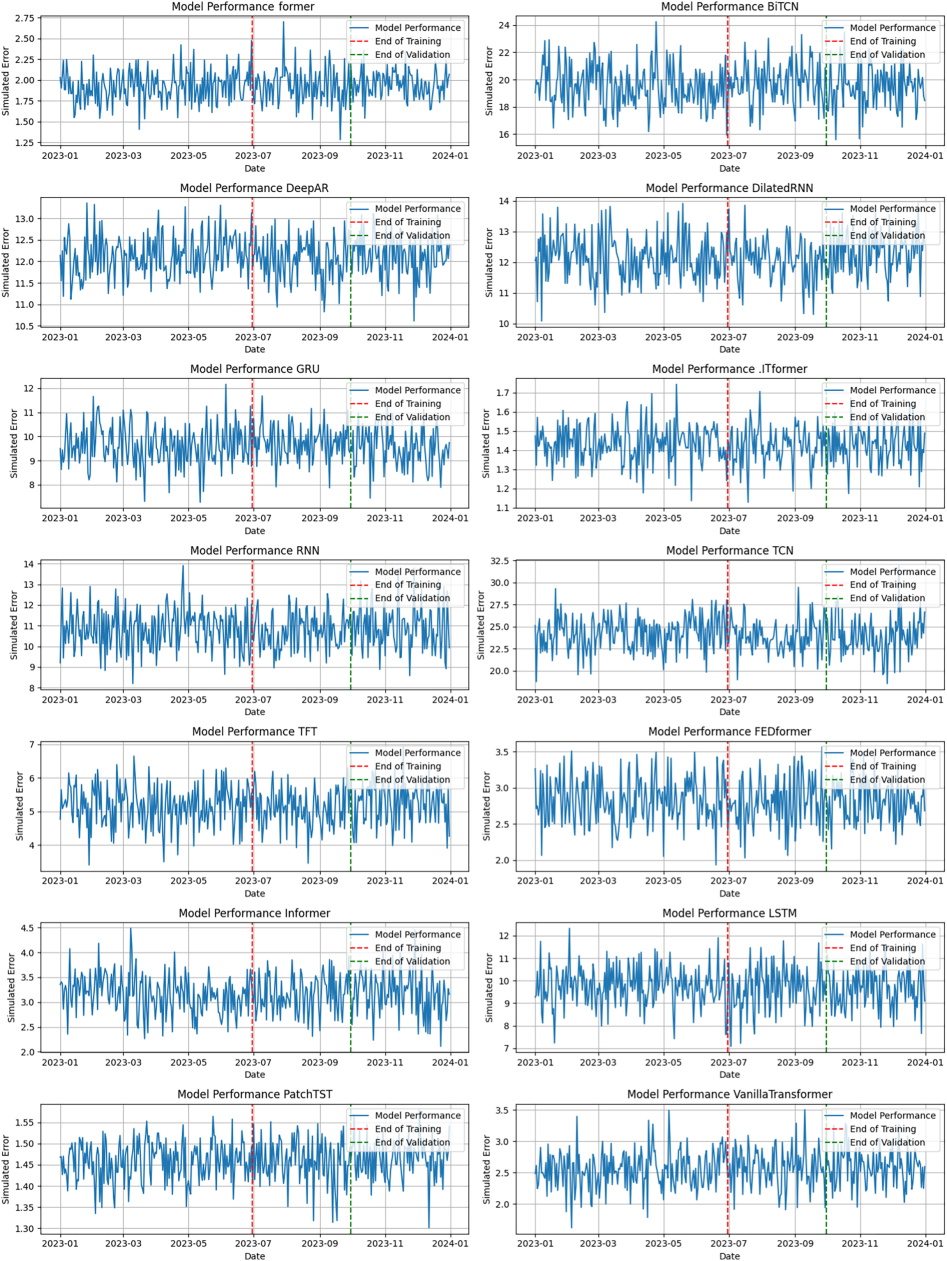
Performance of forecasting models.

Figure 11 illustrates the temporal performance of the models applied to the data. Each subgraph shows the demonstrated error behavior of each model, with the blue line representing performance, while the red and green dotted lines indicate the end of the training and validation period. This structure allows for a precise comparative analysis between the models.

The training period focuses on calibrating the models to identify patterns in historical data, while the validation period assesses their ability to generalize. In this context, models such as PatchTST and Informer stand out for their stability and consistency throughout the transition, showing greater accuracy in their predictions. Models based on transformer architectures, such as Former, FEDformer, and VanillaTransformer, show superior performance, with low errors and greater consistency throughout the year. This reflects their effectiveness in capturing long-term patterns and dependencies, making them ideal for applications that require reliable forecasts over longer time horizons.

On the other hand, models such as BiTCN and TCN show greater error variability, especially during the validation period. This instability indicates difficulties in dealing with complex weather patterns, limiting their applicability in scenarios that require greater precision and stability. On the other hand, models based on RNNs, such as LSTM and DilatedRNN, exhibit intermediate performance. Although they capture temporal dependencies, their greater variability in errors, when compared to transformers, points to challenges in maintaining accuracy over longer time horizons. Finally, the analysis presented in Fig. 11 confirms the advantage of transformer-based models in terms of accuracy and predictive stability. These results provide a basis for selecting models best suited to the demands of weather forecasting, considering the complexity of the data and the resources available.

## Conclusions

The models analyzed were applied to a data set from a local weather station, made up of hourly measurements over a year. These architectures, which include RNNs and transformers, demonstrated different capacities for capturing complex patterns, seasonal variability, and forecast horizons. The analysis revealed that each model has specific advantages and limitations, depending on the metric considered and the intrinsic characteristics of the data, highlighting the importance of aligning the choice of model with the objectives and needs of the application.

Transformer-based models such as Informer, iTransformer, Former, and PatchTST demonstrated superior accuracy in capturing long-term dependencies within weather data, particularly on multi-day forecasts. iTransformer, for instance, achieved the best overall performance, with a MedianAbsE of 1.21, MeanAbsE of 1.24, MaxAbsE of 2.86, RMSPE of 0.66, and RMSE of 1.43. In contrast, RNN-based architectures like TCN and BiTCN showed stronger performance on short-term forecasts but were more prone to higher variance and outlier errors, often reflected in elevated MaxAbsE values. These results align with prior findings that RNNs struggle with long-range dependencies due to vanishing gradients, while transformers maintain context over extended sequences, reinforcing their suitability for long-term weather prediction.

While transformer-based models such as Former, PatchTST, and iTransformer showed greater stability, especially in metrics related to overall accuracy and control of extreme deviations, recurrence-based approaches such as TCN and RNN showed greater sensitivity to rapid oscillations in the data. Although comparisons between models are common, it is rare to find implementations that treat such different architectures uniformly, integrating customized configurations and providing detailed results based on metrics such as RMSE, RMSPE, MaxAbsE, MeanAbsE, and MedianAbsE. This effort at standardization and practical comparison adds significant value, especially in a dynamic field such as weather time series forecasting.

The inclusion of models such as PatchTST, FEDformer, and Informer, which are still emerging, highlights the innovation of their approach. These models, often analyzed in isolation in theoretical studies, have been integrated here in a practical and comparative environment, highlighting their applicability in real scenarios and promoting a comprehensive analysis of their performance. Finally, the approach implemented demonstrates an understanding of the challenges specific to analyzing meteorological time series from a single scenario. This process, adapted in a personalized way for each model, takes advantage of their respective strengths, consolidating an applied technical methodology. Turning this approach into a reproducible practice represents a significant technical differentiator, especially for applications focused on similar meteorological scenarios.

The methodological standardization and detailed evaluation of the metrics establish a relevant starting point for future research. This approach can be expanded to include additional variables, explore different meteorological scenarios, or integrate new emerging models. In addition, the replicability and transparency promoted in this study open up avenues for practical application in other areas that require accurate time series forecasts, such as renewable energy, precision agriculture, and environmental monitoring. To this end, the codes developed are available in the public repository (dos Santos, 2025), ensuring transparency and accessibility for replication and expansion of the work. In this sense, the repository serves not only as a record of the technical effort handled but also as a basis for developing new solutions.

Researchers and professionals can use this modular structure to explore different scenarios, test new models, or integrate additional variables, broadening applications in the field of time series forecasting. In this work, the default hyperparameters of the models considered were considered. Future work can be handled with a focus on hypertuning, using methods such as grid search, Bayesian optimization, and tree-structured Parzen estimator. In future work, statistical tests can be computed to validate the differences between the models, such as significance tests, and to prove the robustness using the Wilcoxon signed-rank test.

## Supplemental Information

10.7717/peerj-cs.3001/supp-1 Supplemental Information 1 Dataset.

## Additional Information and Declarations

### Data Availability

The following information was supplied regarding data availability:

The models are available at GitHub:

- https://github.com/rps-ifpr/Forecasting_Models_Irrigation

- PEREIRA DOS SANTOS, R. (2025). Forecasting Models for Irrigation Analysis (1.0) [Data set]. Zenodo. https://doi.org/10.5281/zenodo.15686280.

The raw data is available in the Supplemental Files.
